# Monitoring progress towards the elimination of measles in Iran: supporting evidence from 2014 to 2016 by application of measles outbreaks data

**DOI:** 10.1186/s12889-019-7060-2

**Published:** 2019-06-03

**Authors:** Naser Piri, Manoochehr Karami, Leili Tapak, Seyed Mohsen Zahraei, Younes Mohammadi

**Affiliations:** 1 Department of Epidemiology, School of Public Health, Hamadan University of Medical Sciences, Hamadan, Iran; 2 Research Center for Health Sciences, Hamadan University of Medical Sciences, Hamadan, Iran; 3 Modeling of Noncommunicable Diseases Research Center, Hamadan University of Medical Sciences, Hamadan, Iran; 4 Center for Communicable Diseases Control, Ministry of Health & Medical Education, Tehran, Iran; 5 Social Determinants of Health Research Center, Hamadan University of Medical Sciences, Hamadan, Iran

**Keywords:** Measles, Epidemiology, Elimination, Effective reproduction rate, Outbreak

## Abstract

**Background:**

To achieve the goal of measles eradication, all WHO member countries should continuously monitor the status of measles elimination. This work aims to characterize measles outbreaks in Iran from 2014 to 2016 and calculate the effective reproduction number, given that the country has recently eliminated measles.

**Methods:**

Effective Reproduction Number (R) was estimated to achieve the goal of measles elimination using measles related outbreaks data and epidemiological data from the cases linked to imported cases. Three methods were used to estimate R includes (i) proportion of cases imported, (ii) distribution of outbreak size and (iii) distribution of outbreak generations.

**Results:**

Of the 153 outbreaks occurring during the three years of the study, 29 outbreaks (19%) were unknown source, 86% of them were single cases. Estimates of R during the study period by proportion of cases imported were 0.79 (95% CI: 0.73–0.86). Corresponding values for distribution of outbreak size and distribution of outbreak generations methods were R = 0.83 (95% CI: 0.68–0.97) and R = 0.76 (95% CI: 0.54–0.90), respectively.

**Conclusions:**

Estimated values of R represent the important achievement that the outbreaks of measles originating from the indigenous genotype in Iran have been eliminated. Moreover, Iran has also achieved the goal of measles elimination by end of 2015.

**Electronic supplementary material:**

The online version of this article (10.1186/s12889-019-7060-2) contains supplementary material, which is available to authorized users.

## Background

Between 2000 and 2016, 87% of measles incidence and 84% measles related deaths in the world have been reduced following an increase in percentage of vaccination coverage by routine immunization and supplementary immunization activities (SIAs). In the Eastern Mediterranean region Office (EMRO), percentage of vaccination coverage for first dose of measles vaccination (MCV1) and second dose (MCV2) was 77 and 69% in 2016, respectively [1, 2]. Following SIAs program in late of 2003, the Islamic Republic of Iran witnessed a period effect in the trend and a rapid shift in the incidence of measles disease. Incidence of measles from 11,874 in 2000 to 79 cases by the end of 2016 has decreased in Iran, as well. The incidence of measles has declined dramatically with the increase in the percentage of vaccination coverage. According to the reports of World Health Organization (WHO), percentage of vaccination coverage for the first and second opportunity of the measles immunization were more than 99 and 98% in Iran, respectively [3, 4]. Published research shows that genotypes B3 and D8, H1 and D4 were detected between 2010 and 2012 by phylogenetic study in Iran [5].

In summary, R_0_ is a measure of dynamics of infectious diseases among susceptible population. While R indicates dynamics of infectious diseases among not necessarily susceptible population. Estimates of R are useful to know the status of measles elimination which indicates lack of existing endemic measles. The selected model for evaluating the status of measles elimination in Iran was the effective Reproduction Number model (R). This model using epidemiological data from the linked to imported case and related case described the status of measles elimination. Measles elimination indicates lack of measles transmission with endemic source for equal or greater than 1 year in a specified area [6]. Estimates R extracted for all years of study were less than one. This indicates that the Iranian population’s immunity threshold is below the epidemic threshold and measles has been eliminated from indigenous genotype by end of 2015.

In the previous published study, R was estimated as 1 − P (where P was equal to the proportion of cases that were imported) to monitor the target of measles elimination between years 2012 and 2014 [4]. In the present study, besides R method, distribution of outbreak size and distribution of the number of outbreak generations were used to address the status and provide supporting documents on measles elimination. Accordingly, this study aims to characterize measles outbreaks and address the status of measles elimination in Iran from 2014 to 2016.

## Methods

We approached registered data on both measles cases and associated outbreak between 2014 and 2016 from national notifiable measles surveillance system. All of available data on measles cases include demographic characteristic such as age, gender, ethnicity, place of residence (city or village), history of vaccination, history of travel, site of transmission, date of rash onset, source of exposure, importation status, country of exposure, diagnostic status (laboratory confirmed or epidemiologically linked and clinically confirmed), and outcome of the disease. A measles case is defined as imported, importation-related, and unknown-source. [6–9].

A measles outbreak is defined as “occurrence of two or more laboratory-confirmed cases that are temporally related (with dates of rash onset occurring between 7-21 days apart) and epidemiological or virological linked or both”. Our definition of endemic measles transmission is “the existence of any continuous indigenous chain of transmission of measles virus that persists for ≥12month in any defined geographic area” [8, 10].

Three different methods were used to estimate R value using R software and verify achievement of Iran to the goals of measles elimination include (i) proportion of cases imported [11] (ii) distribution of outbreak size and (iii) distribution of outbreak generations [12].

We estimated the magnitude of R by defining *imported case* of measles as a case exposed to measles outside the country during the 7 to 21 days before onset of rash. For cases that were outside the country for only a part of the 7 to 21 day interval prior to rash onset, additional evidence including a thorough investigation of contacts of the case is needed to exclude a local source of infection [13]. During elimination phase, it is expect that 80% of outbreaks have less than 10 confirmed measles [4]. If this goal of outbreak size is achieved and the homogeneity condition of the population is established, it indicates the high level of threshold immunity of the population and possibility of self-limitation of outbreaks without human intervention. We estimated the magnitude of R using distribution of outbreak size. A chain of transmission or chain is defined as the entire series of cases that can be linked to the same source. This includes single-case chains, which are not linked to any other cases. Single-case chain includes only one case without more linked cases. It should be noted that incubation period of measles ranges from 7 to 21 days. As third method to estimate the magnitude of R, we calculated the duration of a chain of transmission as the difference between the dates of disease onset of the first and last cases. If this was 0–6 days, cases were considered as being in the same generation; 7–14 days was considered as 1 generation of spread; 15–24 days was considered as 2 generations; and another generation was added for every extra 10 days [13, 14].

Sensitivity analyses was used to confirm estimated values of R. Briefly, we performed estimates on minimum size and generation of outbreaks.

It should be noted that in context of documentation the goal of measles elimination, we considered relevant assumptions similar to published literature. The first assumption is that the measles indigenous transfer has stopped. The second assumption is that all outbreaks of measles without human intervention are self-limiting. The third assumption is that the distribution of the number of secondary cases arising from the imported cases follows the Poisson’s description of the mean R. The fourth assumption, during the study period the average number of secondary cases infected by the index case, is constant throughout the population. Fifth, The pattern of measles related morbidity and relevant contacts with the index are homogenate with equal chance [12, 13, 15].

STATA, V.12, SPSS, V.21, Mathematica, V11.2.0 and R Software, V3.4.3, were used for statistical analysis including descriptive statistics and estimates of effective reproduction number. R codes for calculating the effective reproductive number have been included in Additional file 1: Appendix 1.

## Results

### Characteristics of outbreaks and measles cases

Of 759 reported cases of measles in Iran between 2014 and 2016, 142 cases (40 outbreaks) in 2014, 538 cases (77 outbreaks) in 2015 and 79 cases (36 outbreaks) in 2016 occurred. In total, 55% of 759 measles cases were female. Majority of measles cases during study period were females include 62% of cases in 2016, followed by 57% in 2014 and 53% in 2015. Between 2014 and 2016, 70, 100, 94% of reported cases had a history of vaccination, respectively. Highest number of outbreaks and related measles cases were occurred in 2015. Sixty seven percent of such outbreaks were linked to imported cases. The highest proportion of imported cases was in year 2014. Except for one case related to Iraq, other cases were related to the country of Afghanistan, located to the East of our country. Our data, in terms of the history of contact with suspect or definitive data, was not complete. Most of the reported cases were between the ages of one and 5 years (Table 1).

**Table 1:** Distribution of confirmed cases of measles based on vaccination history and age groups, 2014–2016

Age group		Vaccinated cases	Unvaccinated cases	Under the age of vaccination	Unknown	Total
< 1 years		7 (0.9%)	68 (9.0%)	155 (20.4%)	6 (0.8%)	235 (31.1%)
1 to 4 years		68 (9.0%)	24 (3.2%)	0 (0%)	8 (1.1%)	100 (13.2%)
5 to 9 years		54 (7.1%)	16 (2.1%)	0 (0%)	18 (2.4%)	88 (11.6%)
10 to 14 years		40 (5.3%)	3 (0.4%)	0 (0%)	14 (1.8%)	57 (7.5%)
15 to 19 years		14 (1.8%)	7 (0.9%)	0 (0%)	21 (2.8%)	42 (5.5%)
20 to 24 years		6 (0.8%)	3 (0.4%)	0 (0%)	12 (1.6%)	21 (2.8%)
25 to 29 years		2 (0.3%)	9 (1.2%)	0 (0%)	7 (0.9%)	18 (2.4%)
30 to 39 years		3 (0.4%)	7 (0.9%)	0 (0%)	15 (2.0%)	25 (3.3%)
40 to 59 years		2 (0.3%)	6 (0.8%)	0 (0%)	17 (2.2%)	25 (3.3%)
60 ≤ years		0 (0.0%)	1 (0.1%)	0 (0%)	2 (0.3%)	3 (0.4%)
Unknown	2015–2016	0 (0%)	0 (0.0%)	0 (0%)	2 (0.3%)	2 (0.3%)
	2014^a^	16 (2.1%)	51 (6.7%)	33 (4.3%)	42 (5.5%)	142 (18.7%)
TOTAL		212 (27.9%)	195 (25.7%)	188 (24.8%)	164 (21.6%)	759 (100%)

^a^Data on age groups of cases in 2014 were not available

### Estimates of R (reproduction number)

R was estimated as 1 − P (where P was equal to the proportion of cases that were imported) as described in the methods section. The proportion of imported cases to all cases, R value, from 2014 to 2016 was 24% (2014), 21% (2015) and 10% (2016). The R value based on the proportion of cases imported for the total of 3 years of study was 0.79 (95% CI: 0.68–0.97).

The magnitude of R based on distribution of outbreak size during study period was 0.83 (95% CI: 0.73–0.86). Of the 153 outbreaks occurring during the 3 years of the study, 29 outbreaks (19%) were unknown source, 86% of them were single cases without more related cases. There were 157 cases (21%) of total cases of imported cases who had acquired measles outside the borders of the Islamic Republic of Iran. Number of imported cases in 2014 were 33, 115 in 2015 and 9 cases in 2016. Moreover, 275 cases (36%) were an epidemiological linked with the imported cases (Table 2).

**Table 2:** Number of chains of measles transmission of each size by identified linked to importation, Iran 2014–2016

Number of cases in chain	2014			2015			2016			2014–2016		
	Linked	Notlinked	Unknown	Linked	Notlinked	Unknown	Linked	Notlinked	Unknown	Linked	Notlinked	Unknown
1	4	8	11	10	22	6	19	2	8	33	32	25
2	1	1	1	3	2	0	3	1	0	7	4	1
3–4	2	1	2	5	4	1	1	0	0	8	5	3
5–9	5	0	0	8	3	0	0	0	0	13	3	0
10–24	4	0	0	5	2	0	1	1	0	10	3	0
25–99	0	0	0	4	2	0	0	0	0	4	2	0
100+	0	0	0	0	0	0	0	0	0	0	0	0
Total outbreaks	**16**	**10**	**14**	**35**	**35**	**7**	**24**	**4**	**8**	**75**	**49**	**29**
Total cases	**109**	**13**	**20**	**362**	**167**	**9**	**40**	**31**	**8**	**511**	**211**	**37**

**Fig. 1:**
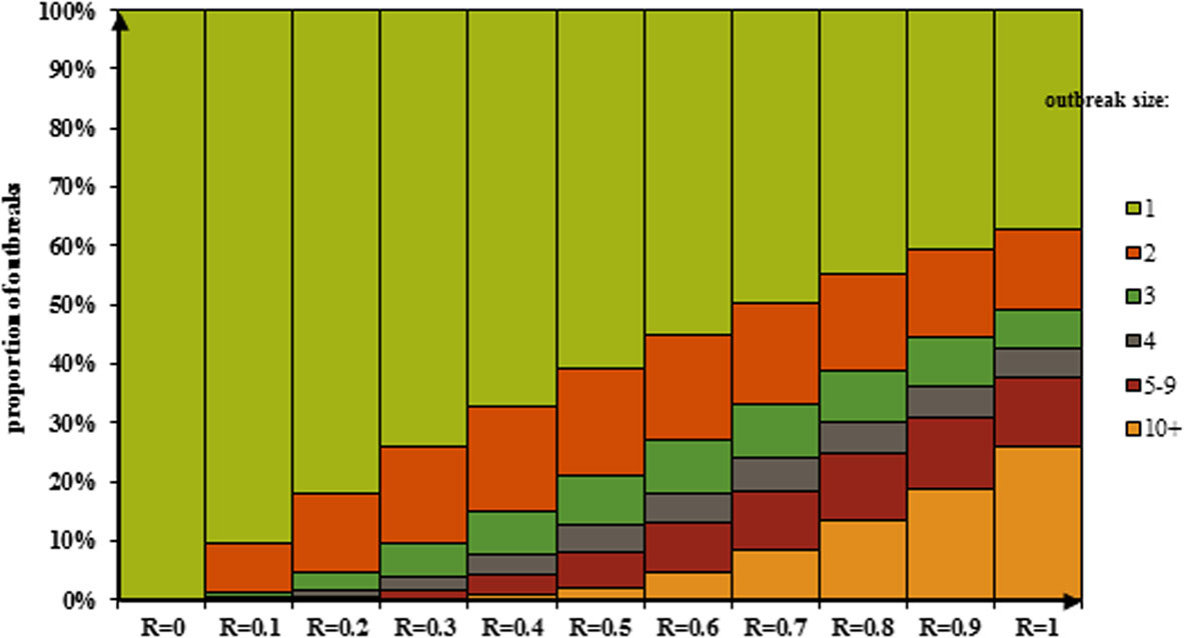
Distribution of the size of measles outbreaks of at least one cases while the first case imported (index cases) to population based on reproduction number

**Fig. 2:**
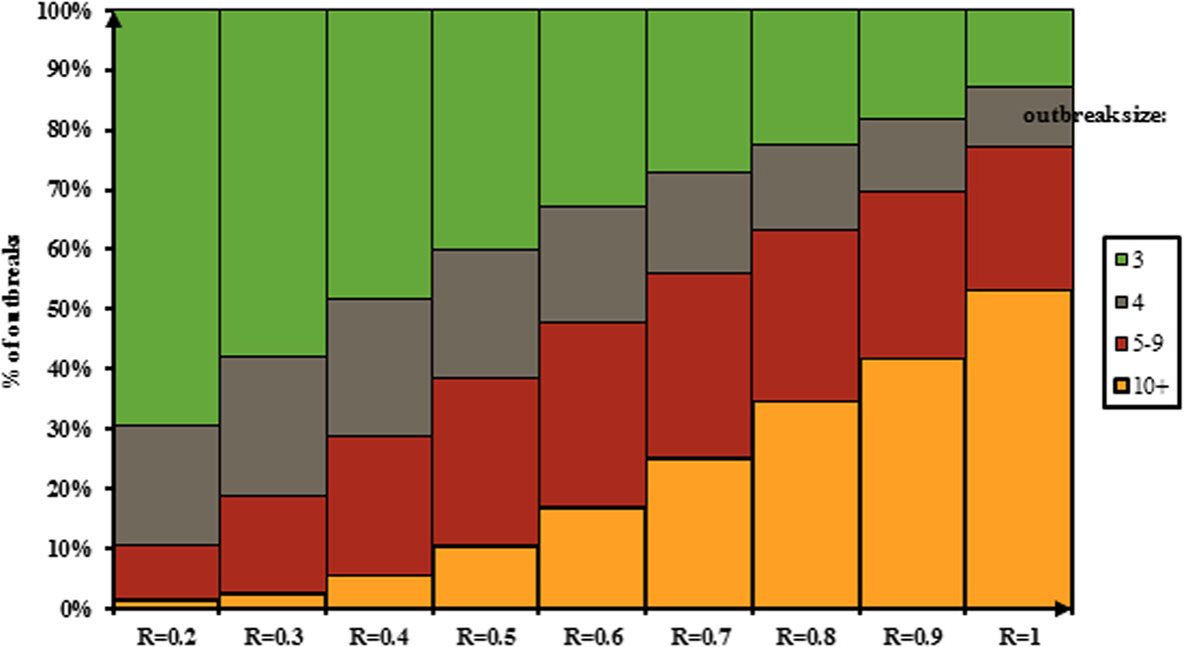
Distribution of the size of measles outbreaks of at least three cases while the first case imported (index cases) to population based on reproduction number

According to the results of this work, distribution of potential outbreak size has been shown based on different range of reproduction number from 0 to 1 in Figs. 1 and 2. Figure 1 shows distribution of outbreaks with at least one cases while the first case imported (index cases) to population. Figure 2 shows distribution of outbreaks with at least three cases while the first case imported (index cases) to population based on reproduction number.

A total of 63 generations of spread have been recorded during the years 2014–2016, 54 generations were ≥ 1 generation of spread. The largest spread of generation related to one of outbreak of 2015 which linked with imported cases that lasted 10 generations. Generations 2 and 4, with the number of 15 and 12 outbreaks, had the highest number of generations, respectively. Seventy six percent of outbreaks’ generations have developed at least four generations within study period. This indicates self-limiting outbreaks caused by the imported case mentioned above (Table 3). The magnitude of R based on distribution of outbreak generation during study period was 0.76 (95% CI: 0.54–0.90) (Table 4).

**Table 3:** Number of chains of measles transmission of each duration (in generations) for the 43 chains involving > 1 case, by identified linked to importation, (Iran 2014–2016)

Generations of spread	2014			2015			2016			2014–2016			
	Linked	Not linked	Unknown	Linked	Not linked	Unknown	Linked	Not linked	Unknown	Linked	Not linked	Unknown	Total
0	1	1	0	2	1	0	3	1	0	**6**	**3**	**0**	**9**
1	1	1	1	0	2	0	0	0	0	**1**	**3**	**1**	**5**
2	4	0	2	6	1	1	1	0	0	**11**	**1**	**3**	**15**
3	4	0	0	1	1	0	0	1	0	**5**	**2**	**0**	**7**
4	2	0	0	6	4	0	0	0	0	**8**	**4**	**0**	**12**
5	0	0	0	2	0	0	0	0	0	**2**	**0**	**0**	**2**
6	0	0	0	4	0	0	1	0	0	**5**	**0**	**0**	**5**
7	0	0	0	2	2	0	0	0	0	**2**	**2**	**0**	**4**
8	0	0	0	1	1	0	0	0	0	**1**	**1**	**0**	**2**
9	0	0	0	0	1	0	0	0	0	**0**	**1**	**0**	**1**
10	0	0	0	1	0	0	0	0	0	**1**	**0**	**0**	**1**
Total	**12**	**2**	**3**	**25**	**13**	**1**	**5**	**2**	**0**	**42**	**17**	**4**	**63**

**Table 4:** Estimates of the reproduction number, R, for measles in the Iran, according to the three estimation of methods, 2014–2016

Method	2014	2015	2016	2014–2016
Proportion of cases imported	0.76 (95% CI:0.62–0.91)	0.79 (95% CI:0.71–0.86)	0.89 (95% CI:0.70–1)	0.79 (95% CI:0.73–0.86)
Distribution of chain sizes	0.72 (95% CI:0.46–0.98)	0.85 (95% CI:0.65–1)	0.69 (95% CI:0.51–0.88)	0.83 (95% CI:0.68–0.97)
Distribution of chain durations	0.55 (95% CI:0.20–0.90)	0.55 (95% CI:0.32–0.78)	0.30 (95% CI:0.00–0.70)	0.76 (95% CI:0.54–0.90)

**Fig. 3:**
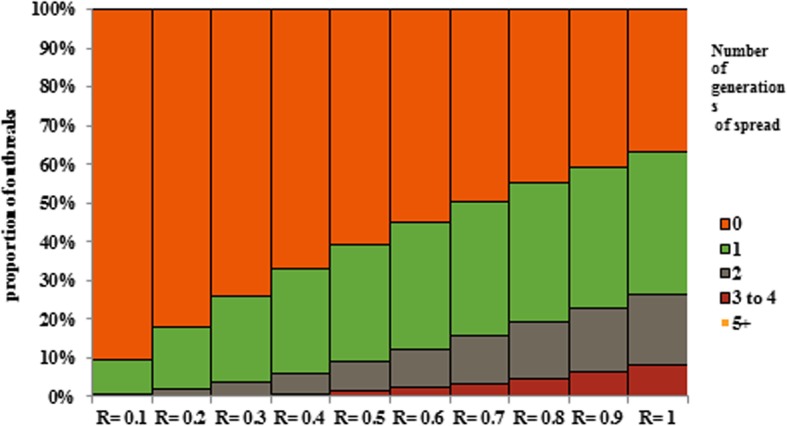
Expected distribution of the duration of chains of transmission produced by an imported case at different values of the effective reproduction number R

Expected distribution of the duration of outbreaks generation following occurrence of an imported case according to different values of the effective reproduction number has been shown in Fig. 3. Many of identified outbreaks in our study were fewer than three cases. To preventing bias and underreporting R values, outbreaks of less than 3 cases and less than 2 generation of spread has been excluded from the analysis.

### Sensitivity analysis

Running sensitivity analysis for the minimum size of outbreaks and outbreaks with an unknown source was performed indicated that R values were slightly increased and that their confidence intervals were wider. However, for minimum generation of spread, the values of R were not significant. However confidence intervals were wider for all generations. The results of the sensitivity analysis for the minimum outbreak size and the minimum generation of spread are not shown here.

## Discussion

Our findings demonstrate the status of measles elimination in Iran using three different methods which used to estimate R value and verify achievement of Iran to the goals of measles elimination. We pointed out interpretation of findings from such three methods include (i) proportion of cases imported (ii) distribution of outbreak size and (iii) distribution of outbreak generations in the remainder of discussion.

In the period of measles elimination and even afterwards, all imported cases, especially primary cases or index cases and linked cases must be identified as soon as possible. Since case-based surveillance system cannot properly identify all imported cases, the R value obtained from proportion of cases imported has some limitations. In countries without a robust surveillance system to identify the imported case, using proportion of cases imported, due to underestimating the value of R, will not certainly indicate the true status of measles elimination.

Measles elimination in the common language, means raising the level of immunity of individuals to the extent that not only measles in that area is eliminated, but by the arrival of an infectious case in a region, nobody would not get infected (or sick). Of 153 outbreaks, 29 outbreaks were unknown source. For 2014 to 2016, 40, 45 and 67% of the outbreaks were linked to the imported case, respectively. Majority of cases were linked to the imported cases. During the years of study, averagely for each year of outbreak, 3 ،5 and 2 people has been infected with measles respectively (Excluding imported cases). The largest outbreaks occurred in 2015, mostly in southern and southeastern parts of the country. Eighty eight percent of the total occurred outbreaks included equal or less than 10 cases and 67% of them were single cases (Table 2). Occurred outbreaks with less than 10 cases represent two very important points: first, it can be indicative of a high level of population immunity and consequently, a reduction in the number of susceptible individuals and those sensitive to indigenous measles virus. On the other hand, it can be a sign of the self-limitation of an outbreak caused by an imported case without human intervention.

Although the goals of the WHO in a number of areas, including EMRO, have not yet been fully met, but remarkable progress has been made, including a 21% reduction in the number of measles cases worldwide (from 50 cases per million in 2010 to 39.3 in 2015). The expected WHO target was ≤5 cases per million populations. According to the WHO reports in 2015, the incidence rate of measles in the EMRO was 33.5 cases per million. This rate for the Islamic Republic of Iran is 5 cases per million. Following the failure to achieve the goal of eliminating measles by the end of 2015, EMR region, the landscape was determined to eliminate measles by the end of 2020 [8, 11, 16].

As a principle, it is recommended that until the eradication of measles throughout the world, national vaccination days, supplementary immunization activities (SIAs), measles routine vaccination with case-based surveillance system and high quality in the Islamic Republic of Iran and other 21 be active, which is both cost-effectiveness and cost-beneficial. Center for communicable diseases control need to conducts field surveys and to collect serological data on measles disease throughout the country annually, especially in the winter and spring, that provides more supported evidence for further research on measles elimination in this way, the amount of R can be estimated using the direct method.

Although before the start of the immunization program, some activities were carried out to control measles disease, since December 6th, 2003, with the launch of the SIAs program, the start of the measles elimination program launched seriously. One year later, the MMR vaccine (measles, rubella, and mumps) was integrated into the country’s EPI program. The target population in the national vaccination plan was all 5 to 25 year olds. The project lasted about 3 weeks. The valuable achievement of this plan was the immunization of 33 million people in the country against three diseases in 2003 [17].

Thirty five percent of cases (268 cases) were people under 1 year of age including 33 cases in 2014, 235 in 2015 and 2016. About 26% (195 cases) had no history of vaccination. Fifty four percent of cases were children under the age of 5 years. Due to the unavailability of age group data for 2014, it is listed in a separate row at the bottom of the Table 1.

The main limitation in this work was outbreaks with unknown source which leaded to failure to identify the imported cases in a many number of outbreaks. Another limitation was lack of data on age of affected patients from measles outbreaks in 2014. This weakness limits the possibility of access to assess the history of vaccination by age.

## Conclusions

Estimated values of R represent the important achievement that the outbreaks of measles originating from the indigenous genotype in the Islamic Republic of Iran have been eliminated. In addition to reaching the fourth millennium development goal (MDG4), Iran has also achieved the goal of measles elimination by end of 2015. However public health authorities should consider large and undocumented immigration from other neighboring countries, especially Afghanistan and Pakistan into Iran, threatens to indigenize their measles genotype in the country and target measles elimination. It is highly recommended to vaccinate both immigrants and refugees from neighboring countries, as well.

## Additional file

Additional file 1: Appendix 1: R codes for calculating the effective reproductive number have been included in the Appendix 1. (DOCX 13 kb)

## Data Availability

Data obtained for this study were not publically available. However, the dataset of the current study is available from the corresponding author at a reasonable request.

## Abbreviations

EMRO: Eastern Mediterranean Region Office
GVAP: Global Vaccine Action Plan
MCV: Measles Containing Vaccine
SIA: Supplementary Immunization Activity
WHO: World Health Organization
