# Supplementary material for: Monitoring progress towards the elimination of measles in Iran: supporting evidence from 2014 to 2016 by application of measles outbreaks data
Source: BMC Public Health. 2019 Jun 3;19:687. doi: 10.1186/s12889-019-7060-2 (PMC6547504; doi:10.1186/s12889-019-7060-2)
Supplement: Supplementary file 1 — Appendix 1: R codes for calculating the effective reproductive number have been included in the Appendix 1. (DOCX 13 kb) [file 12889_2019_7060_MOESM1_ESM.docx]

**Appendix1**

**R codes for calculating the effective reproductive number**

##Enter Data

Sj <- function(R,j){

  R^(j-1)*exp(-R*j)*j^(j-2)/factorial(j-1)

}

Sj_tot <- c()

######################log likelihood

ll <- function(m,R){

  for (i in 1:(length(m)-1)){

    Sj_tot[i] <- Sj(R,i)

    if (m[i]<2) Sj_tot[i] <- 0

  }

  if (sum(Sj_tot) < 1) Sj_tot[(length(Sj_tot)+1)] <- 1-sum(Sj_tot)

  P1 <- sum(m[JJ:length(m)]*log(Sj_tot[JJ:length(m)]))

  P2 <- sum(m[JJ:length(m)])*log(1-sum(Sj_tot[1:(JJ-1)]))

  ll <- P1 - P2

  ll

}

#ll(m,Sj_tot)

#######################deriv

sss <- c()

ssss <- c()

sssss <- c()

dd <- function(m,R){

  for (i in 1:(length(m)-1)){

    Sj_tot[i] <- Sj(R,i)

    if (m[i]<2) Sj_tot[i] <- 0

  }

  if (sum(Sj_tot) < 1) Sj_tot[(length(Sj_tot)+1)] <- 1-sum(Sj_tot)

  for (jj in 1:(length(m))){

    sss[jj] <- exp(jj*R)*jj^(2-jj)*R^(1-jj)*((exp(-jj*R)*(-1+jj)*jj^(-2+jj)*R^(-2+jj))/factorial(-1+jj)-(exp(-jj*R)*jj^(-1+jj)*R^(-1+jj)/(factorial(-1+jj))))*factorial(-1+jj)*m[jj]

    ssss[jj] <- ((exp(-jj*R)*(-1+jj)*jj^(-2+jj)*R^(-2+jj))/factorial(-1+jj)-(exp(-jj*R)*jj^(-1+jj)*R^(-1+jj)/(factorial(-1+jj))))

    sssss[jj] <- (exp(-jj*R)*jj^(-2+jj)*R^(-1+jj))/factorial(-1+jj)

  }

  P2 <- sum(sss[JJ:(length(m))])

  P11 <- sum(ssss[1:JJ-1])*sum(m[JJ:length(m)])

  P12 <- 1-sum(sssss[1:(JJ-1)])

  P1 <- P11/P12

  dd <- P1+P2

  dd

}

#dd(m,Sj_tot,.63,2)

###################second deriv

s1 <- c()

s2 <- c()

s3 <- c()

s4 <- c()

s5 <- c()

hh <-  function(m,R){

  for (i in 1:(length(m)-1)){

    Sj_tot[i] <- Sj(R,i)

    if (m[i]<2) Sj_tot[i] <- 0

  }

  if (sum(Sj_tot) < 1) Sj_tot[(length(Sj_tot)+1)] <- 1-sum(Sj_tot)

  for (jj in 1:(length(m))){

    s1[jj] <- ((exp(-jj*R)*(-1+jj)*jj^(-2+jj)*R^(-2+jj))/factorial(-1+jj)-(exp(-jj*R)*jj^(-1+jj)*R^(-1+jj)/(factorial(-1+jj))))

    s2[jj] <- (exp(-jj*R)*jj^(-2+jj)*R^(-1+jj))/factorial(-1+jj)

    s3[jj] <- (((exp(-jj*R)*(-2+jj)*(-1+jj)*jj^(-2+jj)*R^(-3+jj))/factorial(-1+jj))-(2*exp(-jj*R)*(-1+jj)*jj^(-1+jj)*R^(-2+jj)/(factorial(-1+jj)))+(exp(-jj*R)*jj^(jj)*R^(-1+jj)/(factorial(-1+jj))))

    s4[jj] <- (exp(-jj*R)*jj^(-2+jj)*R^(-1+jj))/factorial(-1+jj)

    s5[jj] <- (exp(jj*R)*(jj)^(3-jj)*R^(1-jj)*(exp(-jj*R)*(-1+jj)*jj^(-2+jj)*R^(-2+jj)-exp(-jj*R)*jj^(-1+jj)*R^(-1+jj))*m[jj]

               +exp(jj*R)*(1-jj)*jj^(2-jj)*R^(-jj)*(exp(-jj*R)*(-1+jj)*jj^(-2+jj)*R^(-2+jj)-exp(-jj*R)*jj^(-1+jj)*R^(-1+jj))*m[jj]

               +exp(jj*R)*jj^(2-jj)*R^(1-jj)*(exp(-jj*R)*(-2+jj)*(-1+jj)*jj^(-2+jj)*R^(-3+jj)-2*exp(-jj*R)*(-1+jj)*jj^(-1+jj)*R^(-2+jj)+exp(-jj*R)*(jj)^jj*R^(-1+jj))*m[jj]

    )

  }

  P1 <- sum(s1[1:(JJ-1)])^2*sum(m[JJ:length(m)])

  P2 <- (1-sum(s2[1:(JJ-1)]))^2

  p11 <- P1/P2

  P3 <- sum(s3[1:(JJ-1)])*sum(m[JJ:length(m)])

  P4 <- (1-sum(s4[1:(JJ-1)]))

  p22 <- P3/P4

  P33 <- sum(s5[JJ:length(m)])

  hh <- P11+p22 +P33

  hh

}

####Newton Raphson method

JJ=2

Rlast=.63

RNew =0

eps <- 1e-15

mydif <- 100

while (mydif>eps){

  RNew <- Rlast- (dd(m,Rlast)/hh(m,Rlast))

  mydif <- abs(RNew-Rlast)

  Rlast <- RNew

  RNew

}

RNew​
